# Competing Sound Sources Reveal Spatial Effects in Cortical Processing

**DOI:** 10.1371/journal.pbio.1001319

**Published:** 2012-05-01

**Authors:** Ross K. Maddox, Cyrus P. Billimoria, Ben P. Perrone, Barbara G. Shinn-Cunningham, Kamal Sen

**Affiliations:** 1 Hearing Research Center, Department of Biomedical Engineering, Boston University, Boston, Massachusetts, United States of America; 2 Center for Biodynamics, Boston University, Boston, Massachusetts, United States of America; 3 Center for Computational Neuroscience and Neural Technology, Boston University, Boston, Massachusetts, United States of America; Cold Spring Harbor, United States of America

## Abstract

Why is spatial tuning in auditory cortex weak, even though location is important to object recognition in natural settings? This question continues to vex neuroscientists focused on linking physiological results to auditory perception. Here we show that the spatial locations of simultaneous, competing sound sources dramatically influence how well neural spike trains recorded from the zebra finch field L (an analog of mammalian primary auditory cortex) encode source identity. We find that the location of a birdsong played in quiet has little effect on the fidelity of the neural encoding of the song. However, when the song is presented along with a masker, spatial effects are pronounced. For each spatial configuration, a subset of neurons encodes song identity more robustly than others. As a result, competing sources from different locations dominate responses of different neural subpopulations, helping to separate neural responses into independent representations. These results help elucidate how cortical processing exploits spatial information to provide a substrate for selective spatial auditory attention.

## Abstract 2

Neurons in the avian auditory forebrain show strong sensitivity to the spatial configuration of two competing sources, even though there is only weak spatial dependence for any single source.

## Author Summary

When a listener is presented with many sound sources at once, it is easier to understand a particular source when it comes from a different spatial location than the other competing sources. However, past studies of auditory cortex generally find that in response to a single sound source, there is not a precise representation of spatial location in the cortex, which makes this effect of spatial location hard to understand. Here, we presented zebra finches with two simultaneous sounds (a birdsong target and a noise masking sound) from distinct spatial locations and recorded neural responses in field L, which is analogous to primary auditory cortex in mammals. When the target sound was presented by itself, the location of the source had little effect on the ability to identify the target song based on neural activity in field L. However, when the target was presented with a masker sound, the location of both sources strongly affected neural discrimination performance. Moreover, different subpopulations of neurons preferentially encoded either target or masker, providing a potential substrate for spatial selective attention. Thus, even though location is not well coded in cortical neurons, spatial information strongly modulates cortical responses.

## Introduction

Past studies of spatial effects in auditory cortex have focused on how spatial location is encoded. These studies typically find that single-unit spatial tuning in cortex is weak [1]–[4], not topographically organized [5],[6], and not encoded independently of other perceptually important features [7]. There is good evidence for a specialized “where” pathway in auditory cortex, in which spatial information plays a larger role than in other cortical areas [8]. However, although we know of no single study that directly compares spatial tuning in cortex to that of lower stages of the auditory pathway, spatial tuning of cortical neurons is generally broad compared to both behavioral sensitivity [4] and spatial encoding in the midbrain [9],[10]. One hint for how to resolve these apparent discrepancies is that in an awake animal performing a spatial task, spatial information in cortical responses is enhanced [10]. Together, these results suggest that although spatial information is available, it is not the primary feature represented in the cortical auditory regions. Instead, spatial information may modulate neural responses in a way that depends on task demands, thus enabling analysis of sound sources in realistic auditory scenes [11],[12].

It may be that spatial effects are not best revealed by looking at how well source location is encoded by neural responses, but rather by examining how source location affects other aspects of information in cortical spike trains. In everyday perception, source location matters most in auditory scenes in which sounds compete with each other. Although listeners can localize a sound source in quiet, this ability is degraded in more typical, real-world settings containing reverberant energy or competing sources [13]. In contrast, in exactly those kinds of realistic situations where there are competing sources, spatial separation helps listeners segregate sounds and enables them to focus selective attention, a critical skill for understanding a source of interest [14],[15]. In this sense, behavioral results support the idea that the locations of competing sources strongly influence auditory perception, regardless of whether the listener can effectively localize in such a setting.

Motivated by these observations, we hypothesized that the effects of spatial location on cortical processing would best be revealed by a study that uses competing sound sources. Rather than focusing on how accurately spatial location of a source was encoded, we explored how competing source locations influenced the ability to encode the identity of a target communication signal (in this case, birdsong). We found that, consistent with our hypothesis, source location of a target song presented in isolation had little effect on how well neurons in avian field L (the analog of mammalian primary auditory cortex [16]) encoded song identity; however, in the presence of a competing noise masker, both target and masker locations strongly influenced encoding of song identity. Moreover, depending on the location of target and masker, different neurons were “best” at encoding identity. Such a coding scheme may provide a substrate for spatial auditory attention, as top-down modulatory control signals could selectively suppress responses of neurons favoring a masker in order to reduce competition and allow more precise analysis of a target from a desired location.

## Results

### Neural Responses Are Sensitive to the Locations of Competing Sources

**Figure 1:**
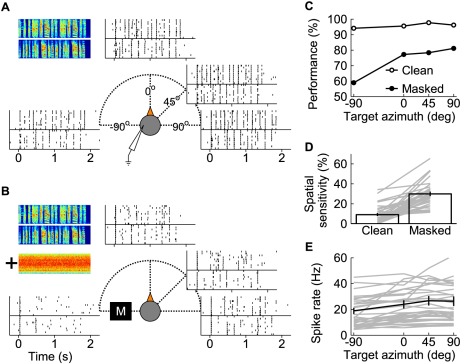
Masking sounds increase spatial sensitivity. (A) Two target song spectrograms (frequency range 500 Hz to 8 kHz), and the response of an example field L recording site to those two songs (10 trials each) as rasters. There is one set of rasters at each of the four azimuths for the two target songs; the effects of changing the location are minimal. (B) The same, with the addition of a song-shaped noise masker (whose spectrogram is shown below those of the targets), played from −90° for all target locations, at the same RMS amplitude as the target (represented by the black box with an “M” on it). The masker sound affects the responses at all target locations, but the effect is stronger (primarily as deleted spikes) when the target is at −90°. (C) Discrimination performance of the same example site. Discrimination of clean targets is reliable for all target locations. However, masked performance is worse when the target is ipsilateral to the site than when it is contralateral. (D) The effect of adding a masker (black bars: means ± 1 SEM, gray lines: individual sites, *n* = 33). The spatial sensitivity is much higher for the masked stimuli, succinctly demonstrating that the addition of masker to a stimulus increases the dependence of performance on location. (E) Average spike rates in response to clean songs (black line: mean ± 1 SEM, gray lines: individual sites).

We recorded neural responses from male zebra finches in the auditory forebrain (field L, based on stereotactic coordinates [17]–[19]) to stimuli from four azimuthal locations in the frontal hemifield. Target stimuli were two conspecific songs, presented either in quiet (“clean”; Figure 1A) or in the presence of a spectrally similar noise masker coming from the same or a different location as the target song (Figure 1B). We assessed neural performance using a single-trial spike-distance-based [20] nearest-neighbor classification scheme [21], calculating a percent correct score that indicates how well neural responses coded stimulus identity. Chance performance was 50%. Consistent with prior studies [17],[22]–[24], rate coding alone was insufficient to allow reliable stimulus discrimination; mean performance when no masker was present was only 54%, averaged across recording sites.

In each experimental session, there were four loudspeaker locations, leading to 16 target-masker spatial configurations. If the recording electrode was in the left hemisphere, loudspeaker locations were on the left side (−90°, ipsilateral to the electrode), in front (0°), halfway between front and right (+45°), and on the right (+90°, contralateral). These locations were flipped about the midline when recording in the right hemisphere. Henceforth, coordinates are referenced to the recording electrode, so that ipsilateral azimuths have negative signs and contralateral azimuths have positive signs. Discrimination performance was calculated for all 16 configurations and three signal-to-noise ratios (SNR; −6 dB, 0 dB, +6 dB). To assess the extent to which the head created an acoustical obstruction (“head shadow”) to the ear opposite the sound source, we measured sound level at both ears from all four locations using a masker token as the probe stimulus. The differences between left and right ears were 1.5, 0.1, −0.8, and −1.3 dB for −90, 0, +45, and +90°, respectively.

For the example site in Figure 1A and B, clean performance was near ceiling at all tested locations. Masked performance was much lower and varied substantially as the target was moved from the ipsilateral side (−90°) to the contralateral side (+90°), holding the masker at −90°. Across recording sites, the masked performance varied much more than clean performance did as a function of location. To quantify this, we computed the spatial sensitivity (defined as the difference between the best and worst performance for a given experimental condition; see Materials and Methods) for each site for both clean and masked targets. Spatial sensitivity was 3-fold higher with a masker present than without (*p*<.001; Figure 1D). The driven spike rate in response to clean songs did not vary significantly with location (*r* = .16, *p* = .068; Figure 1E). This distinction is important: while target azimuth was at best weakly coded by the rate response of the neurons, information about song identity encoded in spike trains varied greatly with target and masker locations.

**Figure 2:**
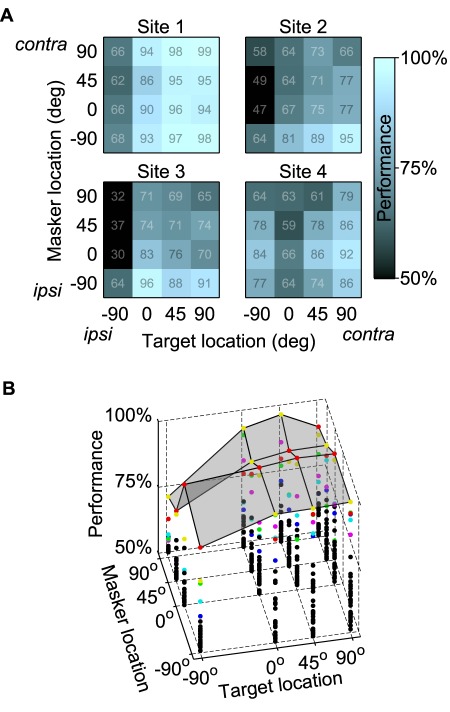
Spatial performance patterns are diverse across neural recording sites. (A) The performances of four example sites that vary widely. The performance is color-coded and percent correct value shown for each spatial configuration. (B) The performances of all neurons at all spatial configurations as dots. The translucent gray shows the “upper envelope”—that is, the surface defined by the best performance across neural sites for each spatial configuration. The best-performing six sites are color-coded so that all the dots of one color show performance for that site for all tested configurations. The order of the colored dots changes across spatial configurations, showing that the diversity of the spatial performance patterns is important for allowing good performance across all spatial configurations. The results shown are for the responses at an SNR of −6 dB; that is, the target sound had half the amplitude of the masker. Recordings were made in both hemispheres and data shown here use the electrode hemisphere as a reference, rather than an absolute left/right coordinate system.

The way in which classification performance varied with spatial configuration varied from site to site. Indeed, some sites responded best when the target was in a particular hemisphere (Figure 2A, site 1), some for a particular target-masker location configuration (sites 2 and 3), and some in idiosyncratic configurations that fit no simple description (site 4).

To explore how such a population of neurons might encode song identity, we considered two population-coding schemes. The first was based on a previous study, which assumed that behavioral performance was determined by the best thresholds across a population of neurons, an approach termed the “lower envelope” principle [25]. Here we define the corresponding neural “upper envelope” as the best classification performance across the entire neuronal population. The performance of individual sites and the upper envelope are shown in Figure 2B as a function of target and masker location for an SNR of −6 dB. While no one site performs well for all spatial configurations, almost all configurations yield at least some sites that encode target identity well.

**Figure 3:**
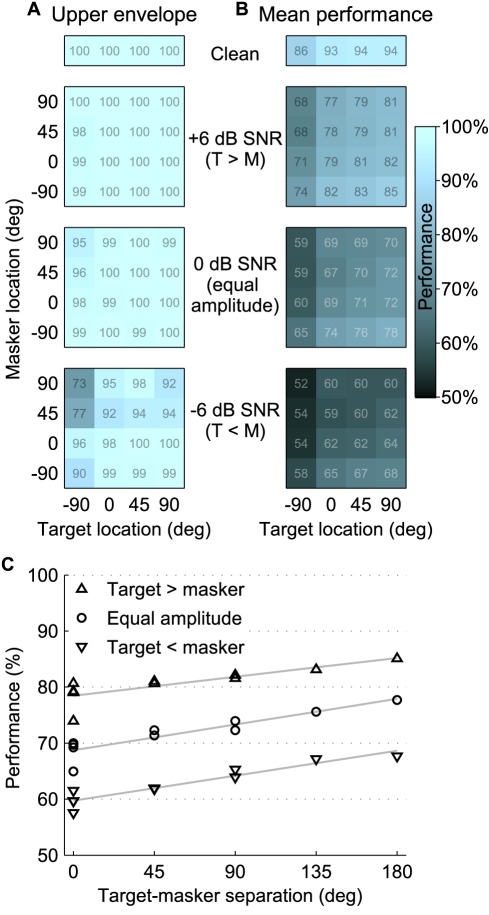
Population measures of response patterns. (A) The upper envelope and (B) the across-site mean performance are shown as grids (with performance color-coded and percent correct value shown in each box) for clean targets and at each SNR (clean, +6 dB, 0 dB, and −6 dB from top to bottom). The upper envelope performance shares its worst-performing spatial configuration with the mean, but is (by necessity) higher than the mean at all points. In fact, the upper envelope performance is at or very near ceiling for the higher two SNRs. At each SNR, the mean varies smoothly as a function of both target and masker location. For both the upper envelope and the mean, the lowest performance is when the target is ipsilateral and the masker is contralateral, and the highest performance is in the complementary configuration. Performance also improves with increasing SNR. (C) Performance increases as a function of spatial separation when considering the subset of spatial configurations in which the target source is more contralateral than or colocated with the masker (in the grids above, the lower right triangle, including the diagonal). Mean performances are shown as markers (upward triangles, circles, and downward triangles for +6, 0, and −6 dB SNR, respectively). Linear regression fits at each SNR are shown as gray lines.

At higher SNRs, the upper envelope is at ceiling (Figure 3A). To better reveal the effects of spatial configuration, we calculated the mean performance across sites for each spatial configuration. Despite the complex dependence of performance on spatial configuration for many of the sites, the mean performance varies smoothly with spatial configuration for each SNR. Specifically, mean performance is best when the target is contralateral and the masker ipsilateral to the neural recording site and worst in the reverse configuration (Figure 3B). Figure 3C shows the mean performances across sites in which the target is farther than the masker from the recording site, in the contralateral direction. Representing the data this way assumes a simple population model in which the neurons in one hemisphere are favored over the other (i.e., the responses from the hemisphere contralateral to the target are enhanced and the ipsilateral responses are suppressed). Using this model (which includes only the values in the lower right half of the grids in Figure 3B, including the diagonal), the effect of spatial separation (as well as SNR) is highly significant (*p*<.001 for both); moreover, linear regression fits at each SNR show that performance improves with increasing spatial separation of target and masker. Such performance increases are parallel with results from behavioral studies in humans [26] and birds [27] that report spatial unmasking.

### Spike Additions and Subtractions

**Figure 4:**
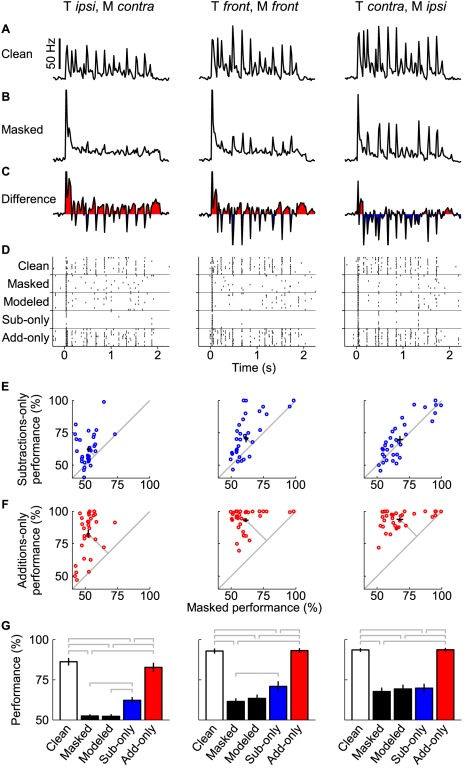
Spike additions and subtractions affect performance differently. For three spatial configurations (left to right: target ipsilateral, masker contralateral; target front, masker front; target contralateral, masker ipsilateral), (A) clean target rate, (B) masked rate, and (C) the difference between masked and clean rates are shown, averaged across all sites. Red peaks show where the masker added spikes, and blue depths show masker subtractions. The large initial peaks have been clipped to increase the dynamic range of the rates that follow. (D) Using these rates, we modeled spike trains that had additions and subtractions, subtractions only, or additions only (“modeled,” which includes additions and subtractions, “sub-only,” and “add-only,” respectively). We calculated percents correct for these generated spike trains for each unit and plotted them against the actual masked performance. (E) The subtractions-only performance for each site (blue circles) and the centroid (black cross, branches are 1 SEM in each direction). Centroids are close to the diagonal, indicating similar subtractions-only and masked performances. (F) Additions-only performances, in the same manner as (E). Centroids are far from the diagonal, indicating a smaller detrimental effect on performance from spike additions. (G) The average (±1 SEM) performance. Additions-only performance does not differ significantly from clean performance for any configuration. Subtractions-only performance is significantly worse than clean performance. As the target moves from ipsilateral to contralateral (and the masker oppositely), subtractions account for an increasing proportion of the masking performance hit, completely accounting for it in the target contralateral, masker ipsilateral configuration. Gray brackets indicate significant differences of *p*<.05.

Maskers degrade responses to target songs. A simple way to evaluate the masker interference is to compare the response elicited by the target in quiet to that of responses to the target plus masker. Differences between the two responses can be categorized into orthogonal categories of spike additions, where the presence of the masker causes extra spikes (usually in the gaps between syllables), and spike subtractions, where spikes that are elicited by the target alone are reduced by the presence of the masker (usually during syllables; see Figure 4A–C). Both types of interference have been studied before [18]; here we extended that analysis. We modeled spike trains that had only subtractions or only additions (Figure 4D; see Materials and Methods), and then calculated performance for these modeled spike trains just as we did for the measured ones.

We first validated our modeling approach by comparing predictions for modeled spike trains containing both additions and subtractions (i.e., the full effect of the masker) to measured data (see Figure 4, “modeled” rasters and performances). The example model rasters look similar to the measured masked spike trains, and target song identification performance closely matched performance using the masked spike trains. These results validate our methods for modeling additions and subtractions.

Following validation, we modeled spike trains that included only spike additions or only spike subtractions to separate their relative effects on performance. When modeling spike additions only (i.e., when no subtractions were modeled), target identification was better than for the measured response. On the other hand, performance for subtractions-only spike trains was only slightly better than the measured responses for two of the three configurations. For the target-contralateral, masker-ipsilateral configuration (right column of Figure 4), performance was essentially equal for the subtractions-only and masked spike trains. These results suggest that additions did not impair discrimination performance when the target was contralateral to the recorded site. However, including additions had some impact on the other two configurations. Overall, this analysis shows that the masker degraded performance more by preventing spikes that a clean target would have elicited than by causing additional spikes.

The times at which spikes are likely to be added by the masker tend to occur when the clean response rates are low. This can be quantified by correlating the clean stimulus response rate (Figure 4A) with the rate of subtractions (blue depths in Figure 4C) as a function of time. This correlation is significant and negative, confirming that subtractions reduce spikes the most when the likelihood of a spike in response to the clean stimulus is great (*r* = −.75, *p*<.001). In contrast, the correlation between the time-dependent spike additions (red peaks in Figure 4C) and the clean rate is weak (*r* = .08, *p*<.001). Taken together, these results suggest that the effect of removing spikes from the peaks interferes with target identification more than adding spikes. This holds true even in spatial configurations where the number of spikes added is greater than the number of spikes removed.

## Discussion

### Specific Experimental Paradigms Unveil Inherent Spatial Sensitivity

Here we show that, in quiet, sound source location has only a modest impact on coding of song identity in field L, an analog of auditory cortex [16]. In general, spatial tuning in brainstem is sharper than in cortex, demonstrating that cortical auditory neurons do not directly inherit the already encoded spatial information present in lower centers of the auditory processing stream [1],[28]. However, our results show that the spatial configuration of competing sources strongly affects the coding of those sources' content.

Spatial effects in cortical neurons are far greater when there are competing sounds than when there is only a single source. This observation suggests that spatial information acts to modulate competition between sources, even in an anesthetized preparation. The fact that these effects arise under anesthesia is important because it shows that they are preattentive. Competition between spatially separated sources helps segregate neural responses, so that information about competing sound sources from different locations is concentrated in distinct subpopulations of cortical neurons. Specifically, most neurons preferentially encode information about contralateral sources; however, some neurons show more specific preferences.

Thus, even though location is not directly coded in cortical neurons, spatial information strongly modulates cortical responses. This idea fits with recent results showing that the effects of source location on neurons in cortex are enhanced when an awake animal is engaged in a task requiring a localization response [10]. The degree to which spatial information affects cortical responses changes with intention: depending on the importance of spatial information to the task being undertaken, spatial coding may be either enhanced or weakened. It is possible that inhibition driven by activity in prefrontal cortex (in mammals) or its analog (in avian species as studied here [29]) causes the sharpened tuning observed during spatial tasks [10]; if so, such connections may also be engaged during selective attention tasks to down-regulate responses of neurons preferentially encoding a masking stimulus that is to be ignored or to up-regulate responses of the distinct population of neurons preferentially encoding the target.

In an anesthetized preparation like that tested here, the enhancement of spatial effects due to the presence of a competing source cannot be coming from top-down modulation from executive centers of the brain. Instead, these effects must be the result of neural circuitry that is “hard wired.” It may be that weak spatial tuning, which is not strong enough to cause observable changes in neural responses with changes in the location of a single sound source played alone, causes large effects when there are multiple sources from different positions. The preattentive spatial competition we find provides a substrate to realize selective spatial auditory attention. Once responses to competing sounds are partially segregated through this kind of preattentive, spatially sensitive process, attentional signals, including inhibitory feedback from executive control areas, can enhance the spatial selectivity already present.

### Acoustic Head Shadow Contributes Little to Observed Spatial Effects

In humans, many spatial effects are explained by the fact that the head causes a significant acoustic shadow at many audible frequencies [26],[30]. When competing sound sources come from different azimuthal locations, the SNR at the ear closer to the target will be greater than if the sources were co-located. This kind of “better-ear” effect has nothing to do with neural processing but is a simple consequence of physics. For the human, such effects can be very significant for speech perception, because the head shadow can be 15 dB or more for frequencies important for speech. Thus, although not interesting from a neural processing perspective, these acoustic effects are important for perception.

Here, in the zebra finch, better-ear effects are small. The zebra finch head is diminutive; its width corresponds to only one quarter of the wavelength of the highest frequency present in our bandlimited stimuli (8 kHz). Given that appreciable acoustic interactions only arise when the wavelength of the sound is comparable to or smaller than the size of the physical object in the environment, the stimuli we presented did not contain frequencies high enough to cause large interaural level differences. This bears out in our measurements, which show an amplitude difference between the ears of approximately 1.5 dB when the stimulus is at ±90°. The better-ear effect is thus limited to 3 dB.

Performance in the target contralateral, masker ipsilateral configuration was 16.8% better than performance in the target ipsilateral, masker contralateral configuration, on average. In contrast, the performance benefit of lowering the masker noise level by 6 dB is only 8.8%. Moving the masker and target in space, then, has nearly double the effect on identification performance as a 6 dB increase in SNR. Given that the maximum effect of acoustic head shadow is only 3 dB, the better-ear acoustic effects cannot explain the spatial effects obtained. Moreover, although a better-ear effect may contribute to the processing of natural broadband signals that contain frequencies high enough to interact acoustically with the zebra finch head, it is unlikely to play a major role in the effects observed here, where we used low-pass filtered stimuli.

### Spike Subtractions Have a Greater Impact than Spike Additions

Interference from a masker on the response encoding a target can be broken down into two forms: spike additions (primarily in the gaps between syllables) and spike subtractions (primarily during syllables) [18]. Here, we quantified the effects of spatial configuration on spike additions and subtractions, and then evaluated modeled spike trains to determine the relative impacts of these effects on neural discrimination performance. In general, additions were more likely than subtractions when the target was ipsilateral to the recording site and masker was contralateral (see Figure 4C), while subtractions were the more prevalent form of interference in the reverse configuration. Because additions and subtractions were calculated by comparing the responses at each spatial configuration to responses to the corresponding target-only stimulus, they represent only the effect of the masker on the response, independent of the minor changes that occur due to absolute target location.

By modeling spike trains with only additions or only subtractions, we were able to gauge their effects on performance. Spike subtractions degraded performance at all configurations (in Figure 4C, blue bars are lower than white bars). In contrast, the spike trains with only additive interference coded target song identity nearly as well as the responses in quiet (red bars are nearly the same as white bars). Additions have a modest impact when subtractions are also present; additions-only performance was better than the fully masked responses in some configurations (compare blue and black bars). Subtractions, on the other hand, interfere with encoding of song identity more seriously and consistently across all spatial configurations.

Although this analysis does not reveal the mechanisms by which a masker interferes with coding of a target, it does give some insight into the complex interactions that take place when two competing sounds are present in an environment. For instance, one might expect, a priori, that the presence of an ongoing masker would cause activity to increase overall, so that the stereotypical target response in quiet is hidden amidst added spikes elicited by the masker. Yet, instead, the detrimental effects of the masker come about primarily from suppression of responses to key features in the target; moreover, the influence of the masker on the target response depends on spatial configuration. This pattern of spatial-configuration-dependent suppression of spikes suggests that competing sources, each preferentially encoded by a distinct neural subpopulation, mutually suppress each other, giving rise to enhanced spatial modulation of responses compared to when a single, unchallenged sound source is presented in isolation.

### Spatial Release From Masking

For a given site, song identity coding tended to vary with both the target and masker locations and generally was best when the target was contralateral from the recording electrode and the masker was ipsilateral to it. For a single site to show spatial release from masking, performance for that site should increase monotonically with increasing spatial separation between the sources. Thus, neither any single recording site nor mean performance averaged over all sites (shown in Figure 3B) exhibits spatial release from masking. Similar results have been seen in the midbrain, in inferior colliculus [28], where, as here, single units showed preferences for encoding responses to different sources, depending on the spatial configuration. However, the activity of thousands of forebrain neurons, not just a single unit, combines to govern perception and behavior. As shown in Figure 3, across the population of neurons in forebrain, there are typically neurons contralateral to the target source that encode target identity well. By looking at the mean performance of neurons at recording sites for which the target sound is more contralateral than the masker (or at the best neuron in that population), performance is predicted to improve with spatial separation (see Figure 3C). Thus, the ensemble of responses, even from an anesthetized bird, can explain behavioral spatial unmasking if one assumes a mechanism as simple as attending to neurons in the hemisphere that favors encoding of the target and ignoring those from the opposite hemisphere.

In behavioral experiments, performance improves with increasing separation between target and masker sounds both for speech and for non-speech sounds [26],[31],[32]. As noted above, better-ear acoustics contribute to spatial release from masking for many sounds important to human behavior, such as speech. Indeed, when a target sound is easily distinguished from a masker (such as when a communication signal is played in steady-state noise), better-ear acoustics can fully account for spatial release from masking in human studies. Interestingly, avian studies do not show the same pattern. The amount of spatial release from masking is essentially identical when behaving birds identify target birdsongs embedded in either a chorus of songs that sound qualitatively like the target songs or a steady-state masker (with the same long-term spectral content as the chorus, but has different short-term structure) [27]. This different pattern suggests that humans can segregate a target from a dissimilar masker even when the two sources are near each other in space, rendering spatial cues redundant [33]. In contrast, birds may be less sophisticated in segregating competing sources, relying more heavily on spatial attributes even when target and masker have distinct spectro-temporal content. Regardless, the current results demonstrate how spatial separation of target and masker can support spatial release from masking in those situations where it is observed behaviorally, no matter what species.

## Materials and Methods

### Neural Recordings

All experimental procedures involving animals were done in accordance with the protocol approved by the Boston University Institutional Animal Care and Use Committee. All subjects were male zebra finches (*Taeniopygia guttata*).

Prior to the day of recording, a preparatory surgery was performed. In this surgery, the location of field L was marked as the point 1.2 mm anterior and 1.5 mm lateral of the midsagittal sinus and a headpin was fixed to the skull. On the day of recording, the bird was first placed in a soft cloth restraining jacket in a quiet, dark room. Injections of urethane anesthetic (20%) were administered every half hour in decreasing amounts (starting with 35 µL) until the bird was unresponsive to its head being patted and its foot being squeezed. Once anesthetized, the bird was placed in a stereotactic frame with its head secured by the previously implanted pin. A craniotomy was performed in which an approximately 2 mm square of skull was removed centered about the spot previously marked as field L. Tungsten microelectrodes (FHC, Bowdoin, ME) ranging in impedance from 2 to 4 MΩ were advanced into the brain using a micron-precision stepper motor. Extracellular potentials were amplified at the headstage, bandpassed between 500 and 10,000 Hz, and recorded with a low-noise soundcard at a sampling rate of 44.1 kHz.

### Stimulus Generation and Presentation

Stimuli were constructed from combinations of two different target zebra finch songs and masking noise (see Figure 1A and B for spectrograms), all filtered between 500 and 8,000 Hz. The songs were chosen to have similar durations (∼2 s); they were songs never before heard by the subjects. To generate the masking noise, several songs were concatenated, the discrete Fourier transform computed, the phase randomized uniformly between 0 and 2π (preserving symmetry), and the inverse Fourier transform computed. The result was noise with a magnitude spectrum identical to the average of the spectra of those songs, but with no temporal structure. Ten independent, random tokens of noise were created so that any residual temporal structure was averaged out across repeated presentations. Independent noise tokens were used on each trial instead of using a single, frozen token because individual noise tokens with the same statistics can have drastically different masking effects [34]. Additionally, the use of independent masker tokens better simulates what happens in natural settings, where, over time, a bird repeatedly hears highly stereotyped songs from its familiar colony mates, but hears them in a different background of masking sources each time.

Stimuli were presented using four single-driver loudspeakers in a sound-treated booth (IAC, Winchester, UK) at a sampling rate of 44.1 kHz. Target songs were normalized so that their root-mean-square amplitudes were 72 dB SPL (c-weighted). The loudspeakers were at four locations in the azimuthal plane: ipsilateral to the implanted hemisphere (−90°), in front of the bird (0°), contralateral to the implant (+90°), and at the angle halfway between the front and contralateral angles (+45°). The speaker locations were referenced relative to the recording electrode, with the side ipsilateral to the implant assigned the negative sign.

Each recording session consisted of 10 blocks. In each block, each of the two target songs was played in isolation from all four locations. Additionally, for each target song, 16 target-masker spatial configurations were tested, each at three SNRs. This resulted in 2×(4+4×4×3) = 104 stimuli per block in which targets were present. We also played the masker alone from each location in each block, resulting in a total of 108 stimuli per block. Each of the 10 blocks used a different, independent token of masking noise semi; the order of the stimuli within each block was randomized. Overall, there were 1,080 two-second stimuli presented with 1.5 s between the end of one trial and the beginning of the next, resulting in a recording session that lasted 63 min for each neural site.

### Spike Extraction and Sorting

Extraction of action potentials (spikes) was performed off-line. First, neural traces were thresholded. The recording to 1 ms on either side of each local maximum was windowed out and considered a potential spike. These waveforms were sorted into user-defined template spike waveforms using a correlation-like coefficient:
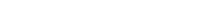
where *x_S_* is a spike waveform and *x_T_* a template waveform, and the sums are taken over time. Spikes were sorted into classes based on the template that yielded the highest *r* or were thrown out if they were not above a minimum *r* to any of the templates. This sorting was verified using principal components analysis clustering. Using this method, single units as well as multiunit clusters (which could not be separated into single units) were extracted. Of the sites that met the minimum performance criterion (see below), 17 were single units and 16 were multiunit clusters. Multiunit activity should produce weaker spatial effects than well-isolated single units. By including both isolated and multiunit recordings in our analysis, our approach is likely to underestimate (if anything) the effects of spatial configuration on neurons in the forebrain. Recordings were made in both hemispheres, but a relative coordinate system was used so that negative azimuths always correspond to the hemisphere ipsilateral the recording site and positive azimuths to the contralateral side.

### Neural Spike Train Analysis

Discrimination performance was calculated using a nearest-neighbor template-matching scheme and a spike distance metric. Methods used were similar to those used in past studies [17]–[19],[22],[24],[35],[36]. To compute pairwise distances between recorded spike trains, each spike train was convolved with a decaying exponential kernel whose time constant determined the effective integration time of the spike comparisons; then the sum of the squared difference was calculated. These distances were used to compare a test spike train against two templates, one from each target song. Each spike train was classified as being elicited by the song whose template was closest to the measured spike train. This process was repeated many times for all spatial configurations for kernel time constants of 1, 4, 16, 63, 251, and 1,000 ms. All but one of the recording sites had an optimal time constant of 16 or 63 ms, in the same range as time constants found in similar past studies (the outlier had an optimal time constant of 251 ms) [17],[22]. In this way, a percent correct score was calculated as a function of time constant, representing how well the spike trains from each spatial configuration matched the target spike trains from the template configuration. The time constant that yielded the best clean target discrimination for each site was used.

Spatial sensitivity was computed as the difference between the maximum and minimum discrimination performance for a given stimulus type. For clean songs, these extrema were determined across the four target locations. For masked stimuli, they were determined across all 16 location configurations, at the SNR that had the highest variance. So that sites with poor performance did not appear spuriously insensitive to spatial configuration, only sites that had an unmasked discrimination performance of 90% or more were included in the analysis.

### Spike Train Modeling

To analyze the effects of spike additions and deletions separately, modeled spike trains were generated. Spike trains were binned into time bins of 2.5 ms. To generate a new spike train, the mean and standard deviation of the number of spikes in each time bin were computed across the 10 responses to each stimulus. Then, the number of spikes in each bin was chosen randomly from a Gaussian distribution with the same mean and standard deviation, with negative spike counts fixed to zero. Time bins in which the masked rate was higher than the clean rate were labeled “additions.” Thus, to simulate a spike train that had only additions, the higher of the masked and clean rates (and the corresponding SD) was chosen at each time bin. Similarly, to simulate a subtractions-only spike train, the minimum of the masked and clean rates was chosen for each bin. The discrimination performance of these simulated spike trains was then calculated in the same manner as real recordings, described above. Refractory period violations (interspike intervals of less than 1 ms) had a negligible effect on analysis.

### Statistical Analysis

The significance of the difference between the spatial sensitivity for clean and masked responses was computed using a paired Student's *t* test. All correlation *r* values were computed using Pearson's product-moment coefficient, with *p* values calculated using a Student's *t* distribution. The significance of spatial separation and SNR for the data shown in Figure 3C were computed using a two-way repeated measures ANOVA. After using a one-way ANOVA to confirm a significant effect of interference type (*p*<.001), Tukey's HSD test was used to compute post hoc comparisons between all performance values at each of the three spatial configurations in Figure 4G. All statistics were done using Matlab's built-in functions. A *p* value of .05 or less was considered significant.

## Abbreviations

SNR: signal-to-noise ratio
